# Wnt Pathway Stabilizes MeCP2 Protein to Repress PPAR-γ in Activation of Hepatic Stellate Cells

**DOI:** 10.1371/journal.pone.0156111

**Published:** 2016-05-23

**Authors:** Soo-Mi Kweon, Feng Chi, Reiichi Higashiyama, Keane Lai, Hidekazu Tsukamoto

**Affiliations:** 1 Southern California Research Center for ALPD and Cirrhosis, Department of Pathology, University of Southern California, Los Angeles, California, United States of America; 2 Department of Veterans Affairs Greater Los Angeles Healthcare System, Los Angeles, California, United States of America; National Institutes of Health, UNITED STATES

## Abstract

PPAR-γ is essential for differentiation of hepatic stellate cells (HSC), and its loss due to epigenetic repression by methyl-CpG binding protein 2 (MeCP2) causes HSC myofibroblastic activation mediated in part via Wnt pathway, the key cellular event in liver fibrosis. Decreased miR-132 was previously proposed to promote MeCP2 protein translation for *Ppar-γ* repression in activated HSC (aHSC). The present study aimed to test this notion and to better understand the mechanisms of MeCP2 upregulation in aHSC. MeCP2 protein is increased on day 3 to 7 as HSC become activated in primary culture on plastic, but this is accompanied by increased but not reduced miR-132 or miR-212 which is also expected to target MeCP2 due to its similar sequence with miR-132. The levels of these mRNAs are decreased 40~50% in aHSCs isolated from experimental cholestatic liver fibrosis but increased 6–8 fold in aHSC from hepatotoxic liver fibrosis in rats. Suppression of either or both of miR132 and miR212 with specific anti-miRNA oligonucleotides (anti-oligo), does not affect MeCP2 protein levels in aHSCs. The Wnt antagonist FJ9 which inhibits HSC activation, increases miR-132/miR-212, reduces MeCP2 and its enrichment at 5’ *Ppar-γ* promoter, and restores *Ppar-γ* expression but the anti-oligo do not prevent *Ppar-γ* upregulation. The pan-NADPH oxidase (NOX) inhibitor diphenyleneiodonium (DPI) also reduces both MeCP2 and stabilized non-(S33/S37/Thr41)-phospho β-catenin and reverts aHSC to quiescent cells but do not affect miR-132/miR-212 levels. Wnt antagonism with FJ9 increases MeCP2 protein degradation in cultured HSC, and FJ9-mediated loss of MeCP2 is rescued by leupeptin but not by proteasome and lysozome inhibitors. In conclusion, canonical Wnt pathway increases MeCP2 protein due to protein stability which in turn represses *Ppar-γ* and activates HSC.

## Introduction

Hepatic stellate cells (HSCs) undergo myofibroblastic trans-differentiation upon liver injury, and this cell fate regulation underlies liver fibrosis. Although many mediators and signaling pathways have been disclosed for this process [1, 2], the loss of expression of peroxisome proliferator activated receptor PPAR-γ, the transcription factor essential for HSC differentiation, constitues one of the most critical molecular mechanisms in HSC activation [3, 4]. Previously, suppressed level of miR132 which presumably targets the methyl-CpG binding protein 2 (MeCP2) mRNA, was proposed to undelie MeCP2 upregulation, which consequently represses *Ppar-γ* via recruitment of the co-repressor HP1α and HDAC at 5’ locus and upregulation of EZH2 methyltransferase which di- and tri-methylate H3K27 at 3’ regions of the gene [5]. This finding is of obvious importance as miR132 may serve as a most upstream molecule for *Ppar-γ* repression in activated HSCs (aHSC) and accordingly as a potential therapeutic target for liver fibrosis.

Activation of NADPH oxidase (NOX) generates superoxide anion in different cell types. In HSCs, NOX1, 2 and 4 are believed to play dominant roles [6–8] and oxidant stress mediated by these NOX isoforms serves as a common signaling event for activation of HSCs induced by key fibrogenic factors such as leptin, angiotensin, PDGF, and TGF-β [9–11]. Morphogens such as wingless-type (Wnt), sonic hedgehog (Shh), and delta-like 1 homolog (DLK1) are also recognized as the mediators of HSC-myofibroblastic cell fate regulation [12–14]. Canonical Wnt pathway activates HSC via MeCP2-mediated *Ppar-γ* epigenetic repression, and this mechanism is also involved in the ability of DLK1 to activate HSC [14]. However, whether the NOX pathway crosstalks with the MeCP2-mediated mechanism for HSC activation is yet to be determined. Moreover, whether and how the Wnt pathway upregulates MeCP2 in aHSC are not known.

The present study examined the roles of miR132 and miR212 which share sequence homology and targets [15] in MeCP2 expression and MeCP2-mediated *Ppar-γ* regulation in Wnt- and NOX-mediated activation of HSCs. In contrary to previous report, our results failed to validate the roles of miR132 or miR212 in MeCP2 regulation in HSC. As seen in HSC with canonical Wnt pathway inhibition, the pan-NADPH oxidase (NOX) inhibitor diphenyleneiodonium (DPI) reduces MeCP2 and non-phospho-β-catenin proteins and restores *Ppar-γ* expression and HSC quiescence but without changes in miR132 and miR212. Finally, reduced MeCP2 protein expression by the Wnt inhibition is due to increased protein degradation, suggesting MeCP2 protein stability is the primary mechanism of its overexpression and consequent epigenetic repression of *Ppar-γ*.

## Materials and Methods

### Primary HSC

HSC were isolated from normal male Wistar rats or rats subjected to 10-day bile duct ligation (BDL) or 6 biweekly injections of CCl_4_ as previously described [14] by the Integrative Liver Cell Core of the Southern California Research Center for ALPD and Cirrhosis.

### FJ9 or DPI treatment of HSC

Primary rat HSC undergoing activation on plastic (cultured for 3 or more days with10% FBS containing low glucose DMEM) were treated with FJ9 (200~600μM), the Wnt antagonist [16], Wnt3a (50ng/ml) or DPI (10 μM) vs. vehicle for 24–72 hr in serum-free medium. Morphological effects were examined by phase contrast microscopy.

### RNA extraction, RT-qPCR, TaqMan qPCR

Total RNA was extracted from HSC using the Quick-RNA MiniPrep (Zymo Research), and cDNAs were synthesized from 1 μg total RNA using the Maxima First Strand Synthesis Kit for RT-qPCR (Thermo Fisher Scientific). Quantitative PCR for *Ppar-γ*, α(I) collagen (*Col1a1*), and α-smooth muscle actin (*Acta2*) was performed by amplifying cDNA for 40 cycles using the SYBR Green PCR master mix (Applied Biosystems); and the ViiA 7 Real-Time PCR System (Applied Biosystems), and the following primers: 5’-CCT GAA GCT CCA AGA ATA CCA AA and 5’ AGA GTT GGG TTT TTT CAG AAT AAT AAGG for *Ppar-γ*; 5’ TCG ATT CAC CTA CAG CAC GC and 5’ GAC TGT CTT GCC CCA AGT TCC for *Col1a1*; 5’-CTG AGC GTG GCT ATT CCT TC and 5’-CCT CTG CAT CCT GTC AGC AA for *Acta2*. Each threshold cycle (Ct) value was first normalized to the 36B4 Ct value of a sample and subsequently to a control sample. TaqMan PCR was performed for matured miR-132, miR212, and miR-137 using pre-designed TaqMan primer/probe sets (Invitrogen^TM^/Thermo Fisher Scientific) and RNA extracted by miRNeasy kit (Qiagen). The data were normalized to 4.5S RNA which has been shown to be minimally affected by experimental conditions.

### Immunoblot analysis

Cell lysates were prepared by washing cells with ice-cold PBS twice, and then lysing on ice for 5 min with RIPA. Samples were assayed for protein concentration using Coomassie (Bradford) Protein Assay Kit (Thermo Scientific), and then mixed with 6x SDS sample buffer before resolving by 6%-10% SDS-PAGE, transferring onto PVDF membrane, and immunoblotting. For immunoblotting, membrane was incubated with anti-MeCP2 (1:1000, Abcam, Sigma Chemical Co.), anti-non-phosphorylated (S33/37/T41) β-catenin (1:1000, Cell Signaling Technology), and anti-β-actin (1:5000, Santa Cruz Biotechnology) antibodies in 5% non-fat-milk/1x TBS containing 0.1% Tween20 overnight at 4°C, followed by incubation with a horseradish peroxidase-conjugated secondary antibody (Santa Cruz Biotechnology). Proteins were detected by a chemiluminescent method using Lumi-Light Western Blotting Substrate kit (Roche), and/or SuperSignal West Femto kit (Pierce). Densimetry of detected bands for protein of interest was performed by using Bio-Rad ChemiDoc^TM^ MP Imaging System and the data were standardized by β-actin density.

### Chromatin immunoprecipitation (ChIP)-qPCR assay

Cross-linking ChIP was performed as previously described [14] using the ChIP-quality anti-MeCP2 antibody (Abcam) and Dynabeads protein A or G (Life Technologies). The thermal cycle was set at 94°C 10 min, followed by 40 cycles of 30 sec at 94°C and 1 min at 60°C for qPCR. A dissociation curve was generated to make sure of the product specificity. The final result was represented as 2^(Ct^_Input_^-Ct^_Output_^)^ and compared to control.

### miR-212/132 promoter activity assay

To assess transcriptional regulation for the miR-212 and miR-132 cluster, promoter deletion-luciferase constructs (-1205/-74, -725/-75, -468/+293, +29/+293) were generated by cloning different lengths of the promoter and regions between the two miRNA sequences into the pGL3-basic vector (Promega) as described [17]. The activated rat HSC line BSC were used for transient transfection with the reporter plasmid as before (13).

### Electroporation for miRNA knockdown and β-catenin expression

To test knockdown effects of miR-132 and miR-212, anti-oligonucleotides for these miRNAs (Invitrogen) were introduced into cultured rat HSC by electroporation using the *Neon*^*TM*^ *Transfection System* (Invitrogen). Using pulse voltage of 1500vol and pulse width of 20ms, more than 80% of day 3 or day 7 cultured HSC were shown to be transduced with a GFP expression plasmid. Using the same method, 3-day HSC were transfected with pBMNz-β-catenin or an empty vector.

### hMeCP2-GFP transduction in HSC and fluorescent microscopy

Primary rat HSC were collected at Day 4, and transfected with the vector pDEST-hMeCP2-GFP (Addgene, #48078) by electroporation as described above. The HSC were then seeded in eight-well chamber slides, treated with FJ9 (600μM) for 72 hours and incubated with different protease inhibitors at indicated concentrations with FJ9 for another 24 hours. The HSC were fixed with 4% paraformaldehyde, blocked with 5.0%BSA, stained with DyLightTM 554 Phalloidin (1:400 dilution, Cell Signailing, #13054S) for 1hour before covering with VECTASHIELD Mounting Medium with DAPI (Vector, H-1200). Then the HSC were imaged using Nikon 5.0 immunofluoresence microscopy.

### Study approval

All animal experiments were approved by the IACUC of the University of Southern California and conducted in accordance with the NIH Guide for the Care and Use of Laboratory Animals.

### Statistical analysis

Data are presented as means ± SD of several experiments. Statistical significance of difference in means was assessed by using the Student's t-test. A p value of less than 0.05 was considered statistically significant and noted with *p<0.05 or **p<0.01 in figures.

## Results and Discussions

### miR-132 and miR-212 levels are not reciprocal with MeCP2 protein expression in activation of HSC

**Fig 1:**
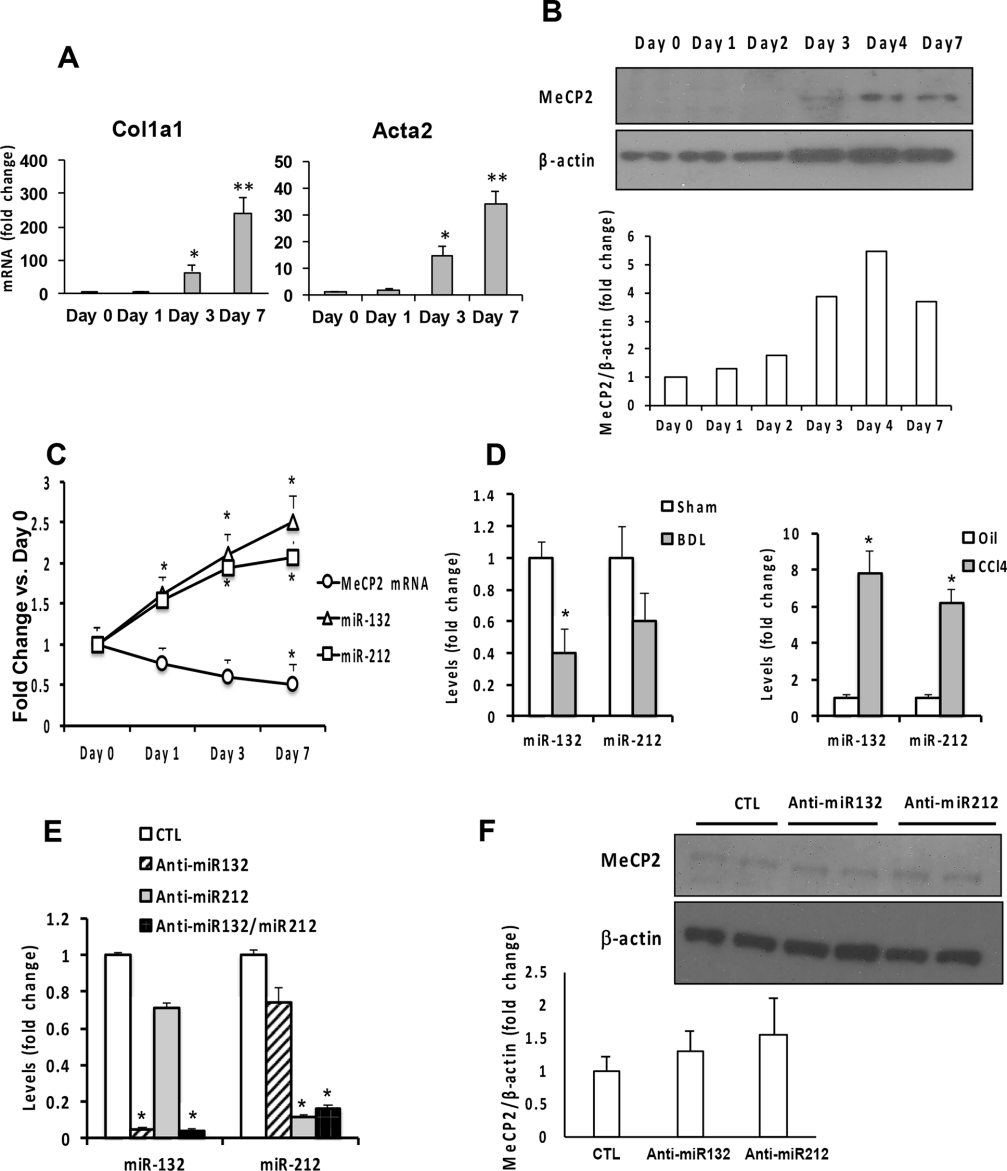
(A) Spontaneous activation of rat primary HSC on plastic dish as demonstrated by conspicuous upregulation of *Col1a1* and *Acta2* mRNA on day 3 and 7 in culture. (B) MeCP2 protein is induced in cultured HSC on day 3–7 as demonstrated by an immunoblot above and densitometry results of fold change of MeCP2 density over β-actin density shown below. (C) MeCP2 mRNA is not induced but reduced in culture-activated HSC. miR-132 and miR212 are upregulated during culture activation of rat primary HSC. *p<0.05 compared to Day 0 values. (D) miR-132 and miR212 levels are reduced in activated HSC isolated from the cholestatic liver fibrosis (BDL) model but increased in activated HSC isolated from hepatotoxic liver fibrosis (CCl4) model as compared to HSC isolated from respective controls (Sham and Oil). *p<0.05 compared to respective control. (E) Transfection of anti-miR132 and ani-miR-212 oligonucleotides by electroporation effectively abrogates the expression of respective miRNA in culture-activated HSCs. *p<0.05 compared to the cells transfected with a control oligo (CTL). (F) miR-132 or miR-212 knockdown with the anti-oligonucleotide do not significantly affect MeCP2 protein levels as compared to CTL. The fold changes in MeCP2/β-actin density are shown below (n = 3).

miR-132 and miR-212 are highly conserved among vertebrates and share similar sequences, thus are predicted to have common targets [15]. A previous report described that MeCP2 is the target of miR-132 and reduced miR-132 is responsible for upregulation of MeCP2 protein and consequent MeCP2-mediated *Ppar-γ* repression in activation of HSC [5]. To further examine this intriguing mechanism and potential contribution of miR-212, we quantified by TaqMan qPCR, the levels of mature miR-132 and miR212 in parallel with assessment of MeCP2 mRNA and protein expression in rat primary HSC from day 0 to day 7 of culture on plastic. HSC undergo spontaneous activation in this culture condition from day 3 and become fully trans-differentiated into myofibroblastic cells on day 7 as evident by conspicuous inductions of *Col1a1* and *Acta2* mRNA (Fig 1A). MeCP2 protein is not detectable in day 0 to day 2 quiescent HSC but becomes induced in activated HSC (aHSC) on day 3 to day 7 (Fig 1B). In contract, MeCP2 mRNA is not induced but rather gradually reduced to 50% on day 1 to day 7 (Fig 1C). These results confirm the previous finding that MeCP2 is upregulated by translational or posttranslational mechanisms. Using the TaqMan miRNA assay and selecting 4.5S RNA as the most unaffected and reliable house keeping snRNA as opposed to U6B which is drastically affected by experimental conditions, we have determined the levels of miR-132 and miR-212. Our results show miR-132 and miR-212 are increased 50% on day 1 and 2–2.5 fold on day 7 (Fig 1C), in contradiction to the notion that reduced expression of these miRNAs causes MeCP2 translational upregulation. Using the same TaqMan qPCR method, we also measured the levels of these miRNAs in aHSC isolated from fibrotic rat livers produced by common bile duct ligation (BDL) or repetitive injection of CCl4, the standard models for cholestatic and hepatotoxic liver fibrosis. miR-132 and miR212 are reduced 40–60% in aHSC from the BDL model as compared to sham-operated control rats but increased 6–8 fold in aHSCs from the CCl4 model (Fig 1D). Collectively, these results do not support that suppressed expression of these two miRNAs is a consistent feature of aHSCs *in vitro* and *in vivo*.

### Anti-oligonucleotides effectively abrogate miR-132 and miR-212 but do not reduce MeCP2 protein in aHSC

We then transfected anti-miR-132 or/and anti-miR212 oligonucleotides (oligos) into cultured aHSC to determine their effects on MeCP2 protein expression. These oligonucleotides effectively suppressed the expression of respective miRNA (Fig 1E) but had no significant effects on MeCP2 protein levels (Fig 1F), suggesting that miR-132 and miR-212 do not regulate MeCP2 expression.

### Wnt antagonism with FJ9 reduces MeCP2 protein and upregulates *Ppar-γ* in a manner independent of miR-132 and miR-212

**Fig 2:**
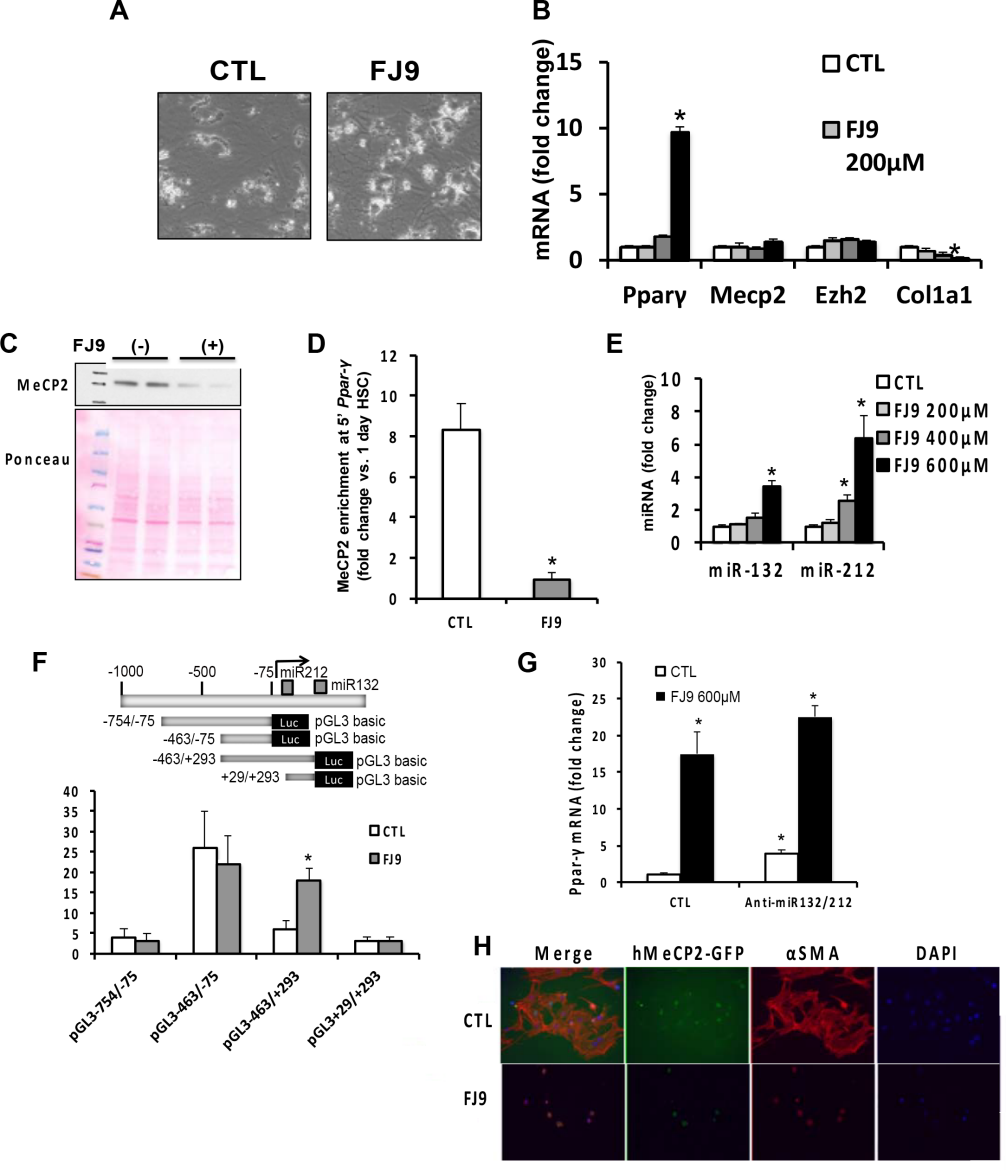
(A) Rat primary HSC cultured for 4 days and treated with a vehicle for 2 additional days (CTL) show the morphology of activated HSC (largely spread cell cytoplasm with reduced lipids). The cells treated with FJ9 (400μM) for 2 days show retracted cytoplasm with dendrite-like processes and increased intracellular lipids (typical morphology of quiescent HSC). Phase contrast microscopy, x100. (B) FJ9 treatment with the increasing concentration as described above, upregulates *Ppar-γ* mRNA and suppresses *Col1a1* mRNA but has no effects on *Mecp2* and *Ezh2* mRNA. *p<0.05 vs. CTL. (C) and (D) The FJ9 treatment (600μM for 48 hr) reduces MeCP2 protein and MeCP2 enrichment to *5’ Ppar-γ* promoter in culture-activated rat HSC. MeCP2 enrichment data are expressed as fold change compared to that in 1 day quiescent HSC which is set at the value of 1. *p<0.05 vs. CTL. (E) The FJ9 treatment as described above increases both miR-132 and miR-212. *p<0.05 vs. CTL. (F) The rat HSC line, BSC, was transiently transfected with the promoter deletion-luciferase constructs as depicted in an upper panel, and treated with FJ9 (400μM for 24 hr). Note a 3-fold increase in the transcription activity with the plasmid with the -463/+293 fragment driving luciferase. *p<0.05. (G) miR-132/miR-212 knockdown with the anti-oligonucleotides as described above, do not affect *Ppar-γ* mRNA upregulation achieved by FJ9 but increases the transcript in non-FJ9-treated cells. *p<0.05 compared to the cells transfected with the control oligo and treated with a vehicle. (H) Rat primary HSC cultured for 4 days were electroporated to transfect the vector expressing hMeCP2-GFP, re-plated for additional 72 hr of culture with FJ9 (600μM) or vehicle. The cells were fixed, stained for F-actin and nuclei with Phalloidin and DAPI, respectively, and examined under fluorescent microscopy.

We have previously shown that Wnt3a treatment increases MeCP2 enrichment to the 3’ *Ppar-γ* promoter to repress *Ppar-γ* expression in aHSC, and Necdin or DLK1 induces MeCP2-mediaetd *Ppar-γ* repression via Wnt pathway [14, 18], placing the Wnt pathway as a converging point for this epigenetic regulation. We aimed to validate this notion by using the small molecule inhibitor FJ9 which interferes the interaction of the Frizzled Wnt receptor and Dishevelled and thus blocks canonical Wnt pathway [16]. The treatment of cultured rat HSC with FJ9 increases *Ppar-γ* mRNA, suppresses HSC activation markers such as *Col1a1* and reverts aHSC to quiescent cells (Fig 2A and 2B). These effects are associated with reduced MeCP2 protein (Fig 2C) and diminished MeCP2 enrichment to *5’ Papr-γ* promoter (Fig 2D). FJ9 treatment increases both miR-132 and miR-212 (Fig 2E). To examine this inductive effect on miR-132/miR-212 at transcriptional level, we tested the HSC line BSC transfected with the plasmid which expresses luciferase downstream of four deletion fragments of the promoter and the region between the two miRNA sequences as depicted in Fig 2F. FJ9 has no effects on luciferase expression by these constructs except that driven by the -463/+293 fragment which shows a significant 3-fold increase (Fig 2F). As the -463/-75 and +29/+293 regions show no effects, these results indicate a region between -75/ and +29 contains a site of Wnt-mediated transcriptional upregulation. To assess the causal link between induced miR-132/miR212 and MeCP2-mediated *Ppar-γ* upregulation, we transfected anti-miR-132 and anti-miR-212 oigos in FJ9-treated HSC. However, this manipulation fails to block *Ppar-γ* upregulation caused by FJ-9 (Fig 2G), suggesting no causal roles of these miRNAs in MeCP2-mediated *Ppar-γ* regulation in these cells. We noticed that *Ppar-γ* mRNA is increased by the anti-miRNA oligos in non-FJ9-treated cells (Fig 2G). As these anti-miRNA oligos do not affect MeCP2 protein (Fig 1F) and the effect is not seen in FJ9-treated (Wnt inhibited) cells, this regulation of *Ppar-γ* mRNA may be mediated by other unknown targets in a Wnt dependent manner.

To further confirm FJ9-induced loss of MeCP2 protein, we overexpressed human MeCP2-GFP fusion protein (hMeCP2-GFP) via a vector in HSC by transfection using electroporation. After transduction, hMeCP2-GFP was mainly localized in nuclei of aHSC with abundant expression of F-actin as demonstrated by fluorescent microscopy. The treatment with FJP for 72 hr reduced hMeCP2 expression as HSC reverted to quiescent cells with abolished F-actin expression (Fig 2H), demonstrating the expression of the human MeCP2 protein is also lost by Wnt inhibition with FJ9. Next, we tested whether overexpression of β-catenin upregulates MeCP2 protein. For this analysis, 3-day cultured HSC were electroporated with an expression vector (pBMNz.β-catenin) or empty vector (pBMNz) and examined MeCP2 protein 3 days later. The expression vector increased the level of β-catenin 2.8 fold and MeCP2 protein 2.5 fold per densitometry analysis (S1 Fig), supporting the positive regulation of MeCP2 protein by the Wnt-β-catenin pathway.

### NOX inhibition with DPI reduces MeCP2 and non-phosphorylated β-catenin, upregulates *Ppar-γ*, and inactivates HSC without changing miR-132 and miR-212

**Fig 3:**
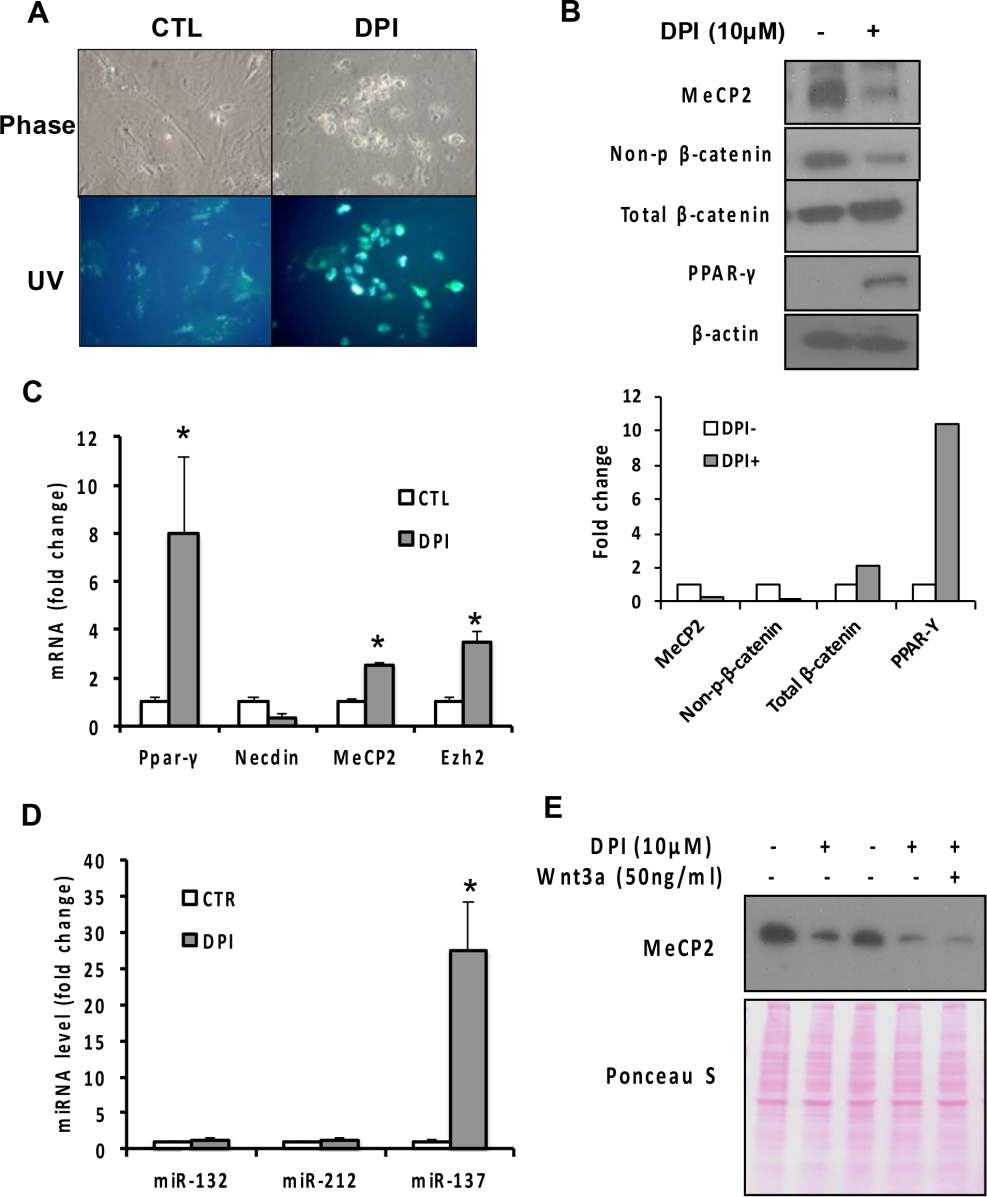
(A) The treatment of culture-activated rat HSC on day 5 with DPI (10μM, 24 hr) reverts activated cells to quiescent cells with increased UV-excited vitamin A fluorescence. (B) The DPI treatment reduces MeCP2 and non-phospho(S30/S37/T41)-β-catenin and upregulates PPAR-γ protein in cultured rat HSC. Densitometric results are shown below. (C) The DPI treatment above upregulates *Ppar-γ* mRNA while suppressing the activation marker *Necdin*. *Mecp2* and *Ezh2* mRNA are also increased. *p<0.05 compared to CTL (vehicle-treated). (D) The DPI treatment has no effects on miR-132 or miR-212 while upregulating miR-137 which was suggested to target *Ezh2*. *p<0.05 compared to CTL. (E) The treatment with Wnt3a does not rescue DPI-mediated suppression of MeCP2 protein. Ponceau S staining shown below demonstrates equal protein loading.

NOX-derived reactive oxygen species mediates HSC activation evoked by different fibrogenic mediators. We examined whether this common signaling pathway is also linked to MeCP2 upregulation. Indeed, the treatment of cultured aHSC with the pan-NOX inhibitor DPI, reduces MeCP2 protein, upregulates PPAR-γ and achieves HSC quiescence (Fig 3A–3C). MeCP2 mRNA is increased by DPI (Fig 3C) despite MeCP2 protein repression, supporting again translational or post-translation regulation of MeCP2. Interestingly, the level of stabilized, non-phospho(S33/S37/T41) β-catenin was also reduced, suggesting DPI’s effects are mediated by Wnt pathway inhibition. However, the levels of neither miR-132 nor miR-212 are changed by DPI (Fig 3D), suggesting the observed effect on MeCP2 is independent of these miRNAs. The DPI treatment conspicuously increases miR-137 (Fig 3D) which was previously suggested to target Ezh2 [19, 20], the gene that encodes a histone methyltrasferase capable of catalyzing H3K27me2/3 modification as a component of the Polycomb Repressive Complex 2. However, Ezh2 mRNA is increased 3-fold by DPI (Fig 3C), suggesting that the miR-137- Ezh2 regulation is unlikely within the context of our experiment. As our results suggested the association of DPI-mediated repression of MeCP2 protein with Wnt pathway inhibition, we texted whether addition of Wnt3a rescues the MeCP2 effect. However, this treatment fails to prevent MeCP2 repression (Fig 3E).

### MeCP2 protein is destabilized by FJ9 treatment

**Fig 4:**
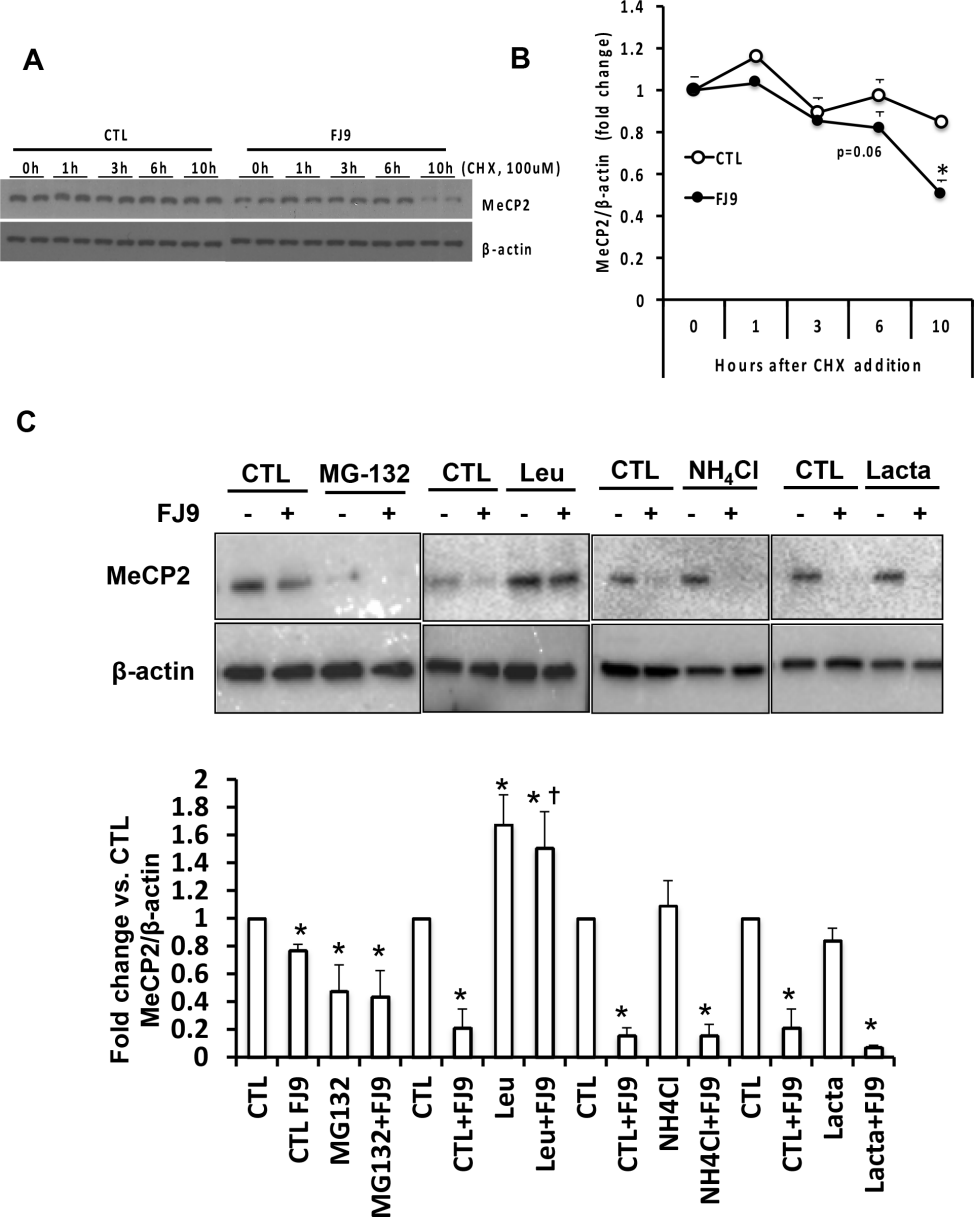
(A) MeCP2 protein stability assay with rat cultured HSC treated with FJ9 or vehicle (CTL) plus cyclohexamide (CHX). (B) Densitometry for MeCP2 and β-actin were performed and the data were expressed as fold change in MeCP2/ β-actin as compared to time 0 (just prior to addition of CHX. *p<0.05 compared to CTL. (C) Reduced MeCP2 protein with FJ9 in rat primary HSC is reversed with leupeptin (Leu), the serine cysteine protease inhibitor but not with proteasome inhibitors, MG-132 and lactacystin (Lacta), or NH_4_Cl which inhibits lysosome activity. Vehicle treatment is designated as CTL. Densitometric data are shown below. *p<0.05 compared to respective CTL (no inhibitor treatment), +P<0.05 compared to CTL cells treated with FJ9 (CTL+FJ9).

MeCP2 protein is known to have a long half-life which is drastically reduced under a variety of conditions including mutations within methyl-CpG binding domain associated with Rett syndrome, a common childhood onset neuro-developmental disorder [21]. We tested whether MeCP2 protein depletion caused by the Wnt antagonism is due to decreased stability. Indeed, the MeCP2 protein stability assay with cycloheximide shows FJ9 treatment destabilizes MeCP2 protein (Fig 4A and 4B). Finally, we tested what proteolytic pathways are responsible for the FJ9-mediated MeCP2 degradation by using MG-132, a reversible proteasome inhibitor, lactacystin, an irreversible proteasome inhibitor, NH4Cl, a lysosomal inhibitor, and leupeptin, a serine and cysteine protease inhibitor. None of these inhibitors but leupeptin prevents the loss of MeCP2 caused by FJ9 (Fig 4C), suggesting that serine and cysteine proteases are causing MeCP2 degradation in FJ9-treated cells and further confirming MeCP2 protein degradation is a primary reason for the FJ9’s effect. We also noticed that MeCP2 expression without FJ9 treatment is increased by leupeptin, suggesting basal expression of MeCP2 protein is also under regulation involving serine and cysteine proteases. MG-132 significantly reduces the basal expression of MeCP2 protein, the effect most likely associated with its known suppressive effects on NF-κB activation via IκB stabilization and HSC activation and survival [22, 23].

In summary, our results do not support the roles of miR-132 and miR-212 in regulating MeCP2 protein in HSC activation in culture or *in vivo*. This obvious discrepancy from the previous report [5] may be caused in part by the different techniques used for analysis of the miRNAs. We have used the TaqMan method with the probes which specifically detect active miR-132 and miR-212 while the miScript Primer Assay (Qiagen) used previously does not offer this specificity and detects both pre-miRNA and mature miRNA. Secondly, we also learned that U6B used previously for the house keeping snRNA is unreliable because it is conspicuously affected by experimental conditions such as HSC activation state as opposed to 4.5S RNA which we determine to be most unaffected and reliable. Using our TaqMan method with 4.5S RNA as the house keeping RNA, the levels of miR-132 and miR-212 are shown not to be reciprocal to MeCP2 protein expression in HSC *in vitro* and *in vivo* models. Further, anti-miRNA oligos which effectively knockdown both miRNAs, fail to regulate MeCP2 protein (Fig 1F) and *Ppar-γ* mRNA (Fig 2G).

But we confirm that inhibition of the Wnt or NOX pathway implicated in HSC activation, is associated with reduction in both MeCP2 protein expression and its enrichment to the *Ppar-γ* promoter and increased *Ppar-γ* expression, validating the central role of MeCP2-mediated regulation in HSC activation. Further, overexpression of β-catenin increases MeCP2 protein in rat primary HSC (S1 Fig), supporting the positive regulation of MeCP2 protein by Wnt- β-catenin pathway. NOX inhibition with DPI causes robust suppression of stabilized β-catenin, suggesting DPI’s effect on MeCP2 may also be mediated via Wnt suppression. However, this effect is not rescued by Wnt3a treatment. This is most likely because NOX’s redox-dependent regulation of Wnt- β-catenin signaling takes place downstream of Wnt-Frz-LRP6 interactions: NOX-derived reactive oxygen species oxidizes nucleoredoxin and prevents it from interacting with Dishevelled, allowing the canonical Wnt pathway to take place [24]. Thus, DPI which inhibits this NOX’s action at the post-receptor level cannot be rescued by the Wnt ligand.

**Fig 5:**
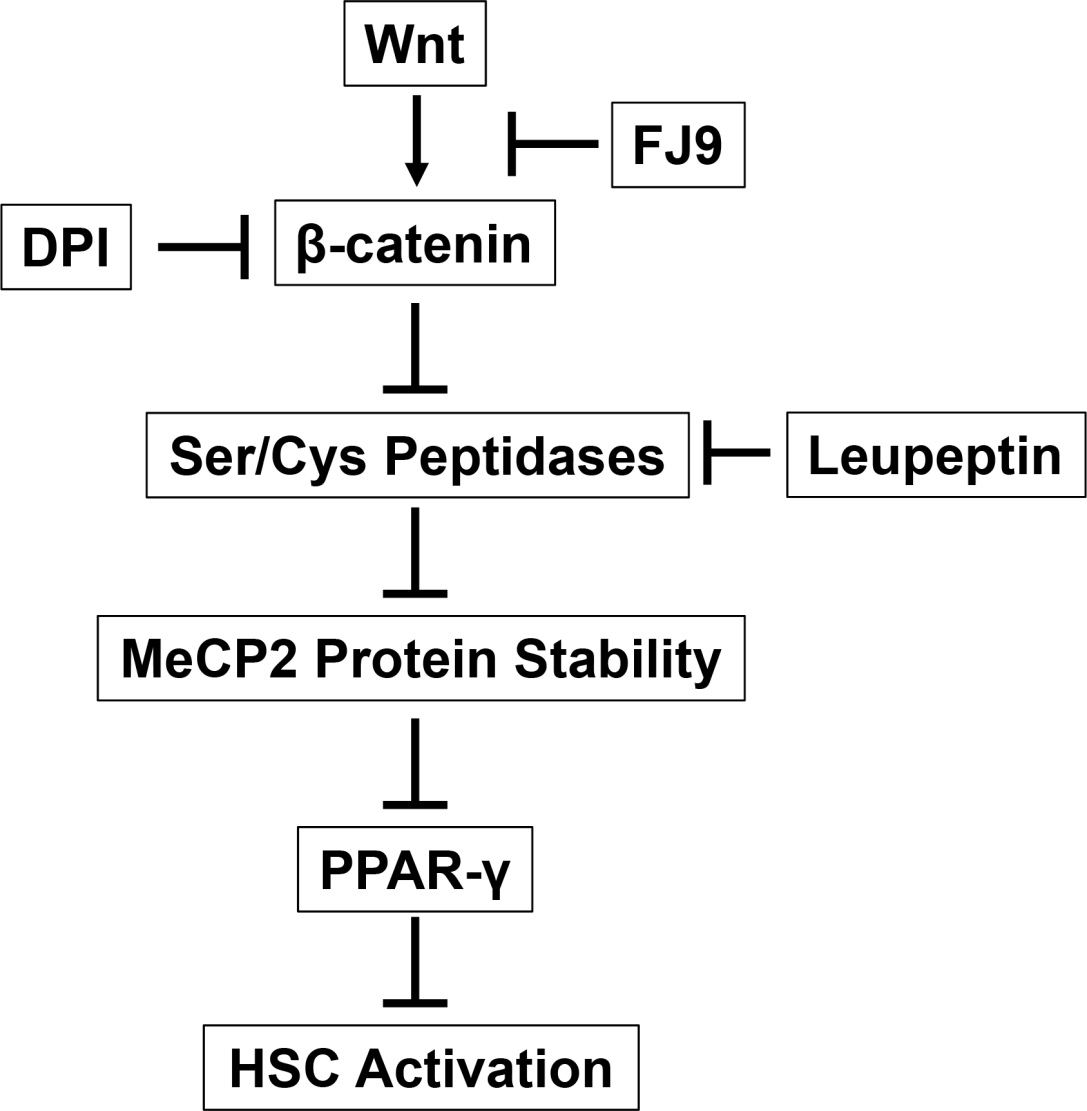
A schematic diagram depicting the proposed Wnt-β-catenin mediated MeCP2 protein stability via regulation of serine and cysteine peptidases in activation of HSC.

MeCP2 degradation is regulated by posttranslational modifications [19] and such modifications may be regulated by components associated with Wnt signaling such as integrin-linked kinase, AKT, and glycogen synthase kinase-β. The primary MeCP2 sequence has two conserved PEST motives enriched in proline, glutamate, serine, and threonine residues and oftern predisposed to rapid proteolytic degradation upon phosphorylation [25]. An unidentified Wnt-inducible gene product may interfere this degradation process at the PEST motives. Our results point to the roles of serine/cysteine proteases in MeCP2 protein degradation which the Wnt pathway inhibits and stabilizes MeCP2 to activate HSC via epigenetic PPAR-γ repression (Fig 5). Expression of serine/cysteine protenases may directly or indirectly be downregulated by the Wnt-β-catenin pathway. Conversely, the inhibitors for these proteases may be positively regulated by the pathway. To this end, it is noteworthy that serine protease inhibitors such plasminogen activator inhibitor-1 (PAI-1) and hepatocyte growth factor activator inhibitor type 1 (HAI-1/SPINT1), are already known transcriptional targets of Wnt-β-catenin [26, 27]. Future studies will address whether and what intracellular serine protease inhibitors capable of stabilizing MeCP2, are positively regulated by the Wnt pathway.

## Supporting Information

S1 Fig Rat primary HSC on day 3 in culture were electroporated with the empty vector (pBMNz) or that expressing β-catenin (pBMNz.β-catenin) and cultured for 3 additional days for protein extraction and immunoblot analysis. Densitometric analysis shows 2.8-fold increase in β-catenin and 2.5-fold increase in MeCP2. Ponceau S staining shows equal protein loading.
(TIF)
