# Supplementary figures and images for: Wnt Pathway Stabilizes MeCP2 Protein to Repress PPAR-γ in Activation of Hepatic Stellate Cells
Source: PLoS One. 2016 May 23;11(5):e0156111. doi: 10.1371/journal.pone.0156111 (PMC4877088; doi:10.1371/journal.pone.0156111)

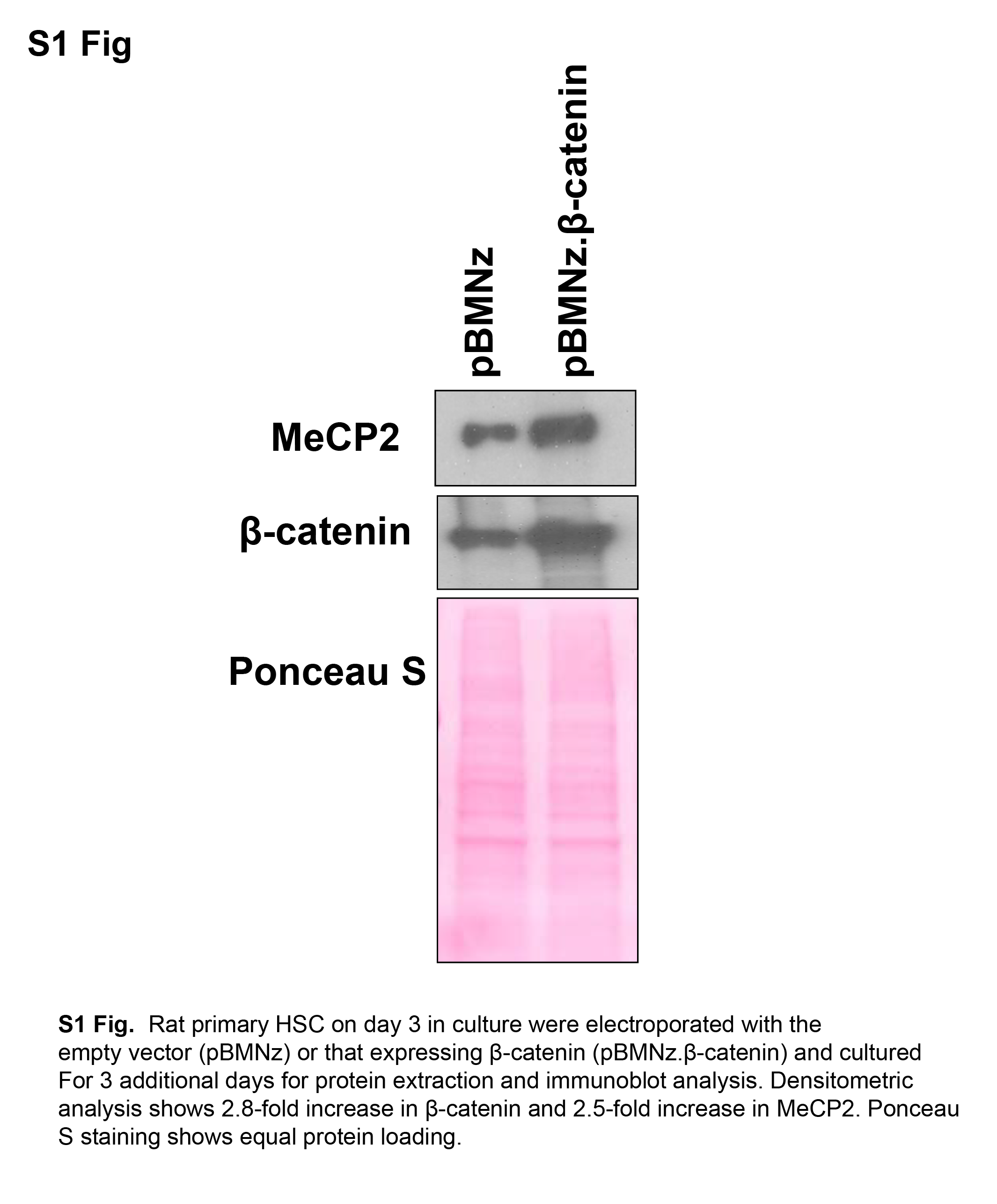

Supplement: S1 Fig — Densitometric analysis shows 2.8-fold increase in β-catenin and 2.5-fold increase in MeCP2. Ponceau S staining shows equal protein loading. (TIF) [file pone.0156111.s001.tif]
